# TRIM31 acts as an intermediate molecule in the process by which Snai2 impairs the proliferation of cervical cancer cells

**DOI:** 10.3389/fonc.2025.1537991

**Published:** 2025-08-22

**Authors:** Nan Cui, Yanru Zhang, Yuan Zhang, Lei Wang, Haiyan Wang, Xiaorui Zhang, Guoqing Tong, Xian Liu

**Affiliations:** ^1^ Department of Reproductive Medicine, The First Affiliated Hospital of Xi’an Jiaotong University, Xi’an, Shaanxi, China; ^2^ Section of Cancer Stem Cell Research, Key Laboratory of Environment and Genes Related to Diseases, Ministry of Education of the People’s Republic of China, Xi’an, Shaanxi, China; ^3^ Reproductive Medicine Center, Ningbo Women and Children’s Hospital of Ningbo University, Ningbo, Zhejiang, China; ^4^ Department of Gynecology, General Hospital of Ningxia Medical University, Yinchuan, Ningxia, China

**Keywords:** Snai2, TRIM31, β-catenin, proliferation, cervical cancer

## Abstract

Snai2 is a transcription factor that inhibits the proliferation of cervical cancer cells and tumor growth. The expression of Snai2 inhibited the expression of β-catenin and impaired Wnt/β-catenin signaling pathway activity. The results of the RNA sequence in Snai2-overexpressing cervical cancer cells implied a strong correlation between Snai2 and TRIM31 with ubiquitin ligase activity. However, the mechanism by which Snai2 regulates TRIM31 remains unclear. In cervical cancer cells, TRIM31 is highly expressed in cervical cancer cells and carcinoma tissues and promotes the proliferation of cervical cancer cells. Furthermore, overexpression or interference with TRIM31 could increase or inhibit the expression of downstream proteins of the classical Wnt signaling pathway, such as β-catenin, cyclin D1 and c-Myc. To the best of our knowledge, rescue of TRIM31 in Snai2-overexpressing cervical cancer cells restored the expression of β-catenin, cyclin D1 and c-Myc. Finally, Snai2 was shown to transcriptionally inhibit the expression of TRIM31 by recognizing and binding to its E-box located in the promoter region. Our findings provide new evidence that TRIM31 may promote cell proliferation and that Snai2 may impair Wnt/β-catenin signaling pathway activity through the transcriptional inhibition of TRIM31. These findings provide new ideas for the regulation of tumor growth and targeted therapy by Snai2-TRIM31 and the Wnt/β-catenin pathway axis.

## Introduction

Cervical cancer is the fourth most common cancer in the world and the second most lethal malignant tumor in developing countries (1). Although the prevention and screening of cervical cancer are becoming increasingly effective, the incidence and mortality rates of cervical cancer are still increasing annually in China, which seriously threatens the health of women in China (2). Currently, cancer stem cells drive the development of tumors and lead to recurrence, metastasis, multidrug resistance, and radiation resistance (3). Many intracellular signaling pathways and factors are involved in the regulation of tumor stemness in cervical cancer cells (4–6).

Snai2 is a member of the highly conserved Snail zinc finger transcription factor family and is involved in a variety of biological processes, including cellular epithelial–mesenchymal transition (EMT) (7). Snai2 plays an important role in promoting metastasis in a variety of tumors. Moreover, it plays dual roles in promoting or inhibiting the proliferation of tumor cells and the self-renewal of cancer stem cells in different tumors. Our previous study revealed that Snai2 was able to inhibit tumors with high EPCAM expression and decrease the stem-cell-like properties of cervical cancer cells. Snai2 negatively regulates the self-renewal of normal hematopoietic stem cells, but the expression of Snai2 in acute myeloid leukemia (AML) promotes the development of leukemia, and the loss of Snai2 expression impairs the self-renewal of leukemia stem cells (LSCs) through the Slc13a3/ROS pathway and significantly prolongs the survival of individuals (8). Snai2 is enriched in basal prostate stem cell (mPSC) populations, and the increased organoid formation capacity of mPSC Snai2^+^ populations mediates TMPRSS4-induced increases in SOX2 and ALDH activity and the acquisition of CSC-like signatures in prostate cancer (9, 10). In addition, Snai2 plays a role in promoting cancer stem cell-like properties in tumor tissues such as head and neck squamous cell carcinoma (HNSCC) and pancreatic cancer (11, 12).

TRIM31 (tripartite motif-containing 31) is a member of the TRIM family with ubiquitin ligase activity, which is involved in a wide range of biological and pathological processes, including autophagy, inflammation, antiviral immunity, and tumorigenesis, by ubiquitinating different substrates and activating a variety of signal transduction pathways (e.g., Wnt/β-catenin, PI3K/Akt) (13). Recent studies have shown that TRIM31 plays a role in promoting tumor progression in a variety of tumors, such as liver cancer, colon cancer, and bladder cancer (14–16). Currently, the role and potential molecular mechanism of TRIM31 in cervical cancer are still unknown.

## Materials and methods

### Tissue specimens and cell lines

All the procedures followed medical ethics approval practices. 40 samples of normal cervical (NC) epithelia and 40 samples of squamous cell carcinoma (SCC) tissue were collected from patients at the First Affiliated Hospital of Xi’an Jiaotong University Medical College from 2015 to 2020. None of the patients had received chemotherapy, immunotherapy or radiotherapy before specimen collection. The human cervical cancer cell lines HeLa, SiHa, and C-33 A were cultured in our laboratory. Dulbecco’s modified Eagle’s medium (DMEM; Sigma Aldrich, USA) was used to culture HeLa, SiHa and C-33 A cells.

Snai2 stably overexpression SiHa-Snai2 cell lines were generated in our previous study. The CMV-SV40-TRIM31 and shRNA for TRIM31 were purchased from Shanghai Genechem (Shanghai, China). Then the CMV-SV40-TRIM31 and shRNA vectors were transfected into C-33 A and SiHa cells with Lipofectamine 2000 reagent (Invitrogen, Carlsbad, CA, USA), and then the cells were treated with G418 (Calbiochem, La Jolla, CA, USA) for three-four weeks to generate stably overexpression and knockdown cell lines.

### RNA preparation and transcriptome resequencing

TRIzol reagent (Invitrogen, Carlsbad, CA, USA) was used to extract the total RNA of SiHa-Vec (n=3) and SiHa-Snai2 (n=3) monoclonal cells in this study for transcriptome resequencing. The BGISEQ-500 platform was used to analyze the samples at the Beijing Genomics Institute (BGI), and the average output of each sample was 22.16 M. The average ratio of sample to genome was 94.15%, and the ratio of comparison to each gene set was 82.37%. A total of 17838 genes were identified in this study. The experimental analysis used the NOISeq method, which is a novel nonparametric approach for the identification of differentially expressed genes (DEGs) based on the thresholds of log2-fold change > 1 and a probability ≥ 0.80, FDR ≤ 0.001. Subsequent data analysis was performed online by Dr. Tom from the Beijing Genomics Institute.

### Western blot

As described in a previous study, the procedure was performed step by step. The primary antibodies against human Oct4 (1:500, sc-5279), Sox2 (1:500, sc-365823), cyclin D1 (1:1000, sc-8396) and GAPDH (1:1000, sc-47724) were purchased from Santa Cruz Biotechnology (Dallas, TX, USA), and TRIM31 (1:1000, YN1699) was purchased from ImmunoWay Biotechnology Company (Plano, TX, USA). β-catenin (1:1000, #8480), Snai2 (1:1000, #9585), and c-myc (1:1000, #8583) were purchased from Cell Signaling Technology (Littleton, CO, USA). The secondary antibodies anti-rabbit or anti-mouse IgG were purchased from Thermo Fisher Scientific (New York, NY, USA). The relative densities of the Western blot bands were measured via the Alpha View system (Cell Biosciences, Santa Clara, CA, USA).

### CCK8 and MTT assays

Cell viability was analyzed with Cell Counting Kit‐8 ((TargetMol, Boston, USA) according to the manufacturer’s protocols. The cells were seeded and cultured at a density of 1000 per well in 100 μL of medium in 96‐well microplates (Corning, USA). Then, the cells were treated with various concentrations of Tan‐I (0, 1.2, 2.4, 4.8 and 9.6 μg/mL). After treatment for 24 h, 10 μL of CCK‐8 reagent was added to each well, and the cells were then cultured for 2 h. All the experiments were performed in triplicate. The absorbance was analyzed at 450 nm via a microplate reader (Bio‐Rad, Hercules, CA, USA) using wells without cells as blanks. The proliferation of the cells was expressed as the absorbance.

For 3-(4,5-dimethylthiazole-2-yl)-2,5-diphenyl tetrazolium bromide (MTT), cells (1000 cells per well) were seeded in 96-well plates (with six parallel samples per condition). The cells were assessed every day (7 days total) via the MTT assay according to standard protocols (Sigma-Aldrich, USA). Briefly, 20 μl of MTT (5 mg/mL) was added to each well and incubated for 4 h at 37°C, followed by the dissolution of 150 μL of dimethyl sulfoxide. The number of live cells was determined by the absorbance at 490 nm (Bio-Rad). Each experiment was performed in triplicate.

### Immunostaining

Both the immunohistochemistry and immunocytochemistry experiments were similar with those in previous studies. TRIM31 (1:100, YN1699) was purchased from ImmunoWay Biotechnology Company. The immunohistochemistry results were examined by two separate researchers via an Olympus CX31 microscope (Olympus, Tokyo, Japan) in five randomly selected representative fields at 40× magnification. The evaluation of TRIM31 staining was performed via the immunoreactivity score (IRS). The score was determined by multiplying the staining intensity by the degree of staining. The intensity of TRIM31 staining was scored as 0 (negative), 1 (weak), 2 (moderate), or 3 (strong). The extent of staining was scored as 0 (0%), 1 (1%-25%), 2 (26%-50%), 3 (51%-75%), or 4 (76%-100%) according to the percentage of positively stained cells. TRIM31 staining was classified into two categories according to the IRS: negative (1-4) and positive (5-12).

### Soft agar colony formation assay

A soft agar assay was used to evaluate the capacity for long-term cell proliferation and survival in 3D culture. The culture mixture containing 0.5% low-melt agarose was plated in the lower layer of the six-well plates and then cultured at 37°C for 4–5 h. The upper layer containing low-melting agarose at a final concentration of 0.3% was mixed with 2000 cells per well, 500 μL of the culture medium was added, and the mixture was subsequently cultured for 2–4 weeks at 37°C. Image J (version 1.48) software was used to count the number of colonies.

### Luciferase reporter assay

For promoter analyses, six fragments (from position -134 bp to -680 bp relative to TRIM31) containing the E-box site of CANNTG were cloned and inserted into the pGL3-Basic vector (Promega, Madison, WI, USA) to generate TRIM31 promoter reporter constructs. Plasmids containing firefly luciferase reporters were cotransfected into SiHa-Snai2 cells and control cells in triplicate via Lipofectamine 2000 (Invitrogen, Carlsbad, CA, USA). The thymidine kinase promoter Renilla luciferase reporter plasmid (pRL-TK) was used as an internal control. The activity of both the firefly and Renilla luciferase reporters was detected 48 h after transfection via a dual-luciferase assay kit (Promega, Madison, WI). The specific promoter activity is presented as the relative ratio of firefly luciferase activity to Renilla luciferase activity. The specific promoter activity is presented as the change in the SiHa-Snai2 group versus the SiHa-GFP group. The primers and oligonucleotides used are listed in the supplemental information, **Supplementary Table S1**. Restriction enzymes were obtained from TaKaRa. All the constructs were verified by sequencing. The specific activity is shown as the fold change in the SiHa-Snai2 group versus the SiHa-GFP group.

### Quantitative chromatin immunoprecipitation

SiHa-Snai2 and SiHa-GFP cells were subjected to ChIP via an EZ-ChIP Assay Kit (Millipore). As described in a previous study, the cells were treated with 37% formaldehyde to crosslink proteins, and the reaction was terminated with 0.125 M glycine. After sonication, the chromatin-protein complexes were immunoprecipitated with 5 μg of anti-Snai2 antibodies (#9585, Cell Signaling Technology) or 1 μg of mouse IgG. Real-time PCR was performed to amplify the regions of interest or internal negative control regions. Each sample was assayed in triplicate, and the fold enrichment ratio was calculated as the value of the ChIP sample versus the corresponding input sample. Samples that yielded twofold enrichment or better were considered positive targets. The primers used for these studies are listed in the supplemental information, **Supplementary Table S2**.

### Statistical analysis

Student’s t test or one-way ANOVA was used for univariate analyses. Statistical analyses were performed via the Statistical Package of Social Science (SPSS) software, version 20.0 (SPSS Inc., Chicago, IL, USA). All the tests were two-sided. A *p* < 0.05 was considered to indicate statistical significance.

## Results

### The expression of TRIM31 is negatively related to Snai2

**Figure 1:**
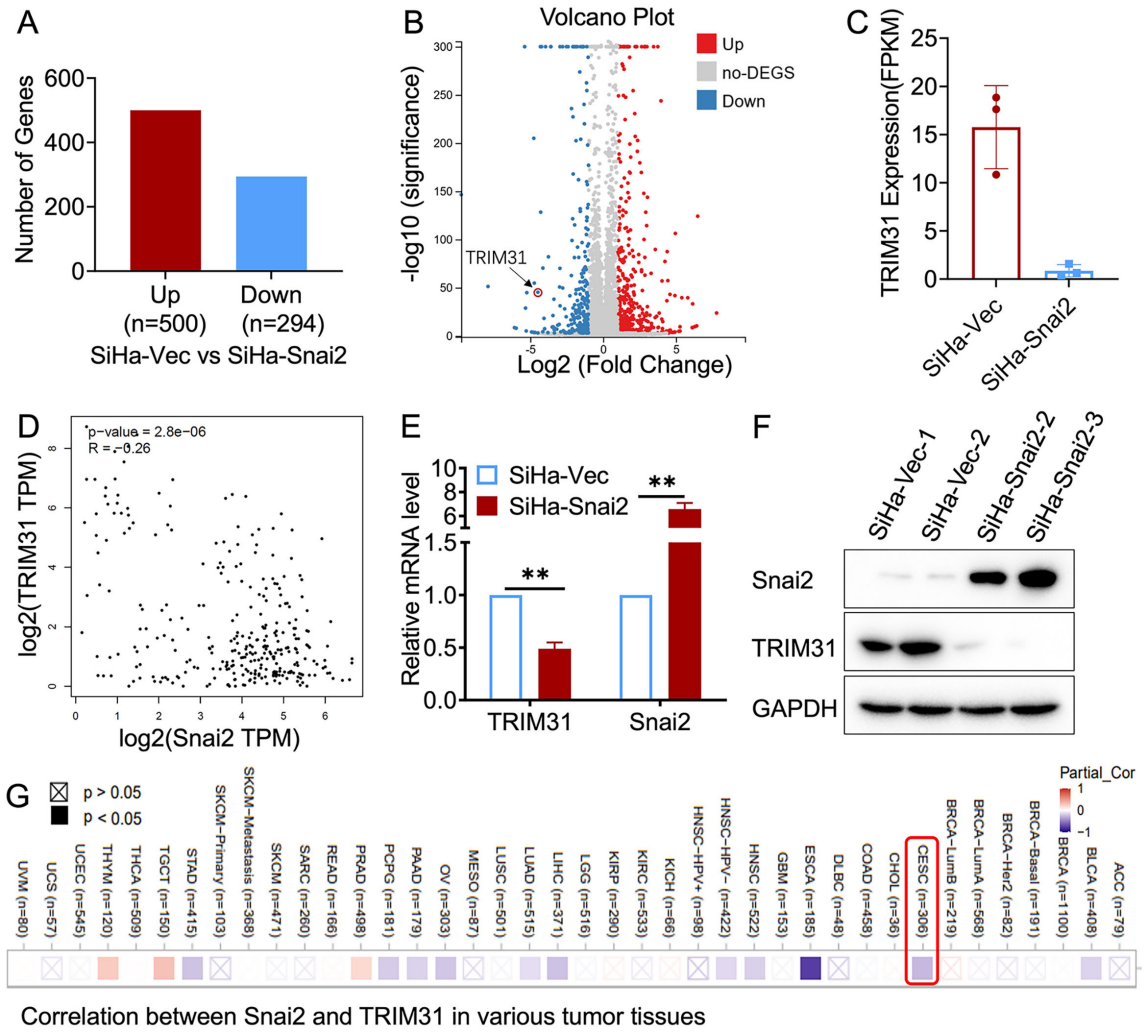
Relationship between TRIM31 and Snai2. **(A)** Differential gene expression by RNA sequencing of SiHa-Snai2 and SiHa-Vec cells is presented as the number of genes **(A)** and volcano plot **(B)**. **(C)** Relative expression of TRIM31 determined by RNA sequencing in SiHa-Snai2 cells compared with SiHa-Vec cells. **(D)** The correlation between Snai2 and TRIM31 in cervical cancer in GEPIA online database. **(E)** The RNA expression level of TRIM31 determined by real-time PCR. **(F)** The protein expression level of TRIM31 in SiHa-Snai2-overexpressing cells and control SiHa cells. **(G)** The correlation between the Snai2 and TRIM31mRNA level in cervical cancer patient tissues was analyzed via TIMER2.0 online database. ** *p* < 0.01.

RNA sequencing was performed in SiHa cells with exogenous Snai2 expression (SiHa-Snai2) and matched control cells (SiHa-Vec). Approximately 294 genes were downregulated and 500 genes were upregulated in SiHa-Snai2 cells compared with SiHa-Vec cells (**Figures 1A, B**). The downregulation of TRIM31 was detected in Snai2-overexpressing SiHa cells (**Figures 1C, D**), which was also confirmed at the mRNA and protein levels (**Figures 1E, F**). An online database (http://gepia.cancer-pku.cn/) of cervical cancer tissue and normal cervical tissue also revealed a negative relationship between TRIM31 and Snai2 (**Figure 1G**).

### Aberrant expression of TRIM31 in cervical cancer

**Figure 2:**
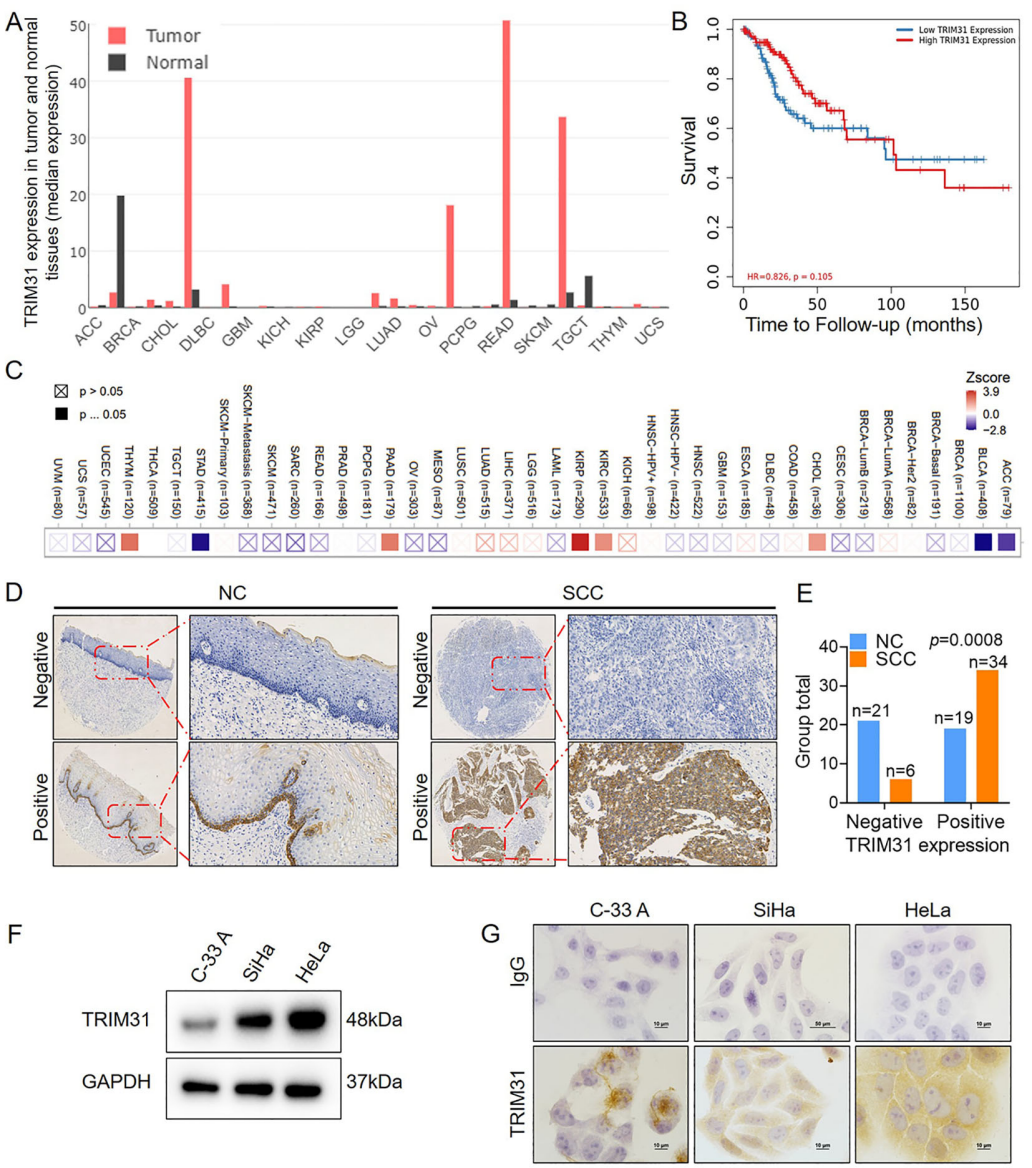
TRIM31 expression in cervical cancer. **(A)** Analysis of the median expression of TRIM31 in a variety of tumor tissues and normal tissues in GEPIA online database. **(B)** Analysis of the effect of the TRIM31 expression on the prognosis of cervical cancer patients in an online database. **(C)** Heatmap between TRIM31 mRNA expression and patient survival in different clinical tumor samples in TIMER2.0 online database. **(D)** Immunohistochemistry of TRIM31 in SCC and NC; original magnification, ×1000. **(E)** TRIM31 staining was classified into 2 categories (negative and positive), and the percentage of each group is shown for 40 NC samples and 40 SCC samples. **(F)** Western blot of TRIM31 in the cervical cancer cell lines C-33 A, SiHa, and HeLa. **(G)** Immunocytochemistry of TRIM31 in the cervical cancer cell lines C-33 A, SiHa, and HeLa.

The level of TRIM31 expression was highly variable across different types of tumors (**Figure 2A**), and the correlation between TRIM31 expression and patient survival in cervical cancer and other multiple tumors was shown in **Figures 2B, C**, according to the online database. Furthermore, the positive expression of TRIM31 in SCC was significantly greater than that in the NC (**Figures 2D, E**). TRIM31 was detected in the C-33 A, SiHa and HeLa cervical cancer cell lines and was highly expressed in SiHa and HeLa cells (**Figures 2F, G**). These results indicated that the expression of TRIM31 was aberrant in cervical cancer cells and cervical cancer tissues.

### TRIM31 enhances stem cell-like properties properties in cervical cancer

**Figure 3:**
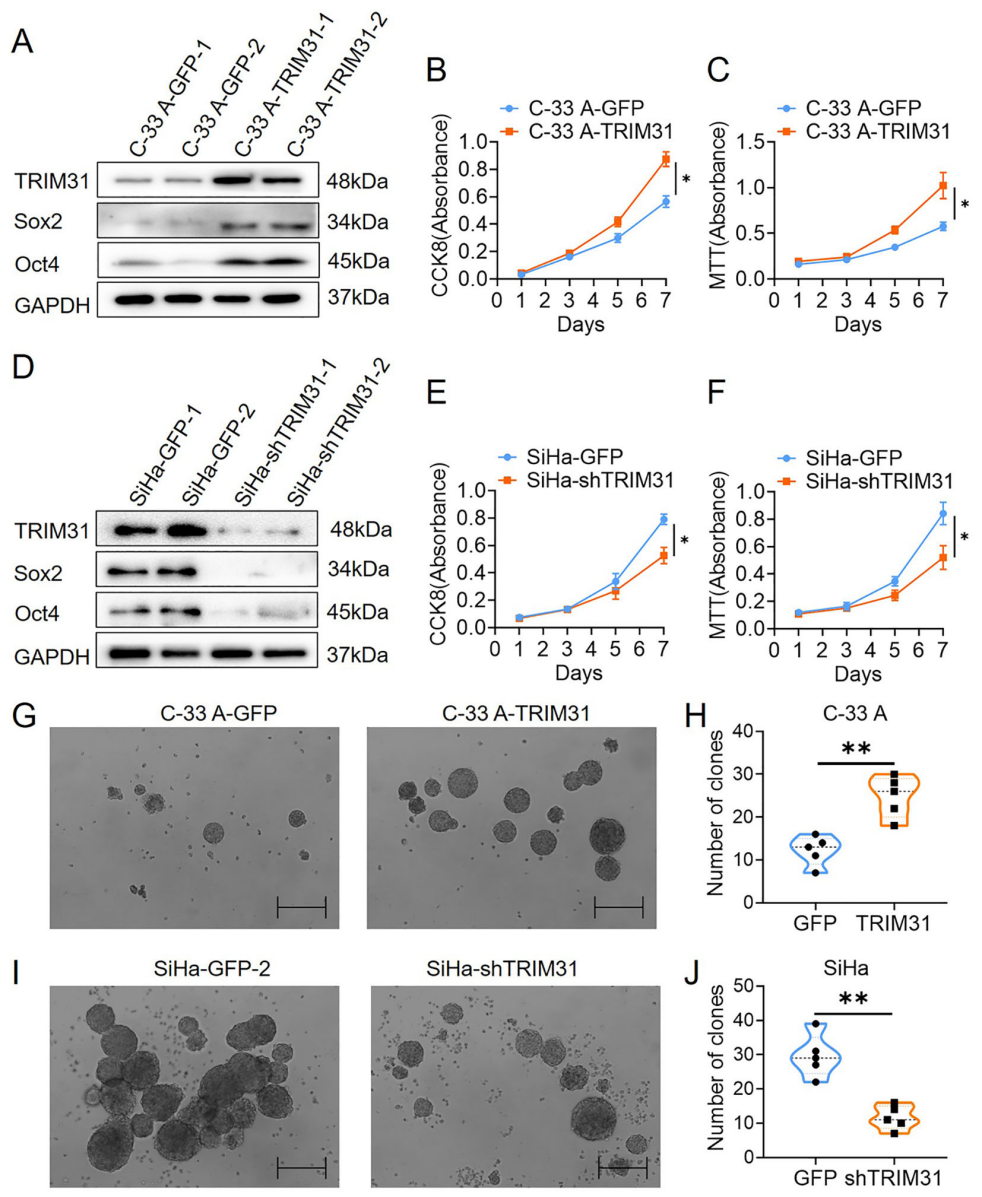
TRIM31 promotes the proliferation of cervical cancer cells. **(A)** Western blotting was used to detect the expression of TRIM31, Sox2 and Oct4 in C-33 A cells (C-33 A-TRIM31) and control C-33 A cells (C-33 A-GFP); CCK8 **(B)** and MTT **(C)** assays were used to detect the effect of the overexpression of TRIM31 on the proliferation of C-33 A cells. **(D)** Western blotting was used to detect the impaired expression of TRIM31, Sox2 and Oct4 in SiHa cells (SiHa-shTRIM31) and control SiHa-TRIM31 cells (SiHa-GFP); CCK8 **(E)** and MTT **(F)** assays were used to detect the effect of impaired expression of TRIM31 on the proliferation of SiHa cells. **(G)** Soft agar colony formation assay of C-33 A-TRIM31 and C-33 A-GFP cells. **(H)** Numbers of clones were calculated between C-33 A-TRIM31 and C-33 A-GFP cells. **(I)** Soft agar colony formation assay of SiHa-shTRIM31 and SiHa-GFP cells. **(J)** Numbers of clones were calculated between SiHa-shTRIM31 and SiHa-GFP cells; *p < 0.05, **p < 0.01.

TRIM31 expression in C-33 A cells was upregulated by stable transfection of a constructed TRIM31-expressing plasmid (C-33 A-TRIM31) (**Figure 3A**) and downregulated in SiHa cells by stable transfection of a shRNA-targeting TRIM31 plasmid (SiHa-shTRIM31) (**Figure 3D**). Compared with those of the control group, the CCK8 and MTT assays revealed an enhancement of the proliferation of C-33 A-TRIM31 cells and a decrease in the proliferation of SiHa-shTRIM31 cells compared with that of the control group (**Figures 3B-F**). In brief, TRIM31 promoted the proliferation of cervical cancer cells, whereas defects in the expression of TRIM31 impaired cervical cancer cell proliferation. More colonies formed in C-33 A-TRIM31 cells than in the corresponding control cells according to the soft agar colony formation assay, whereas fewer colonies formed in SiHa-shTRIM31 cells than in the corresponding control groups (**Figures 3G-J**). Moreover, Sox2 and Oct4 were significantly upregulated in C-33 A-TRIM31 cells compared with those in C-33 A-GFP cells (**Figure 3A**). In contrast, Sox2 and Oct4 were significantly downregulated in SiHa-shTRIM31 cells compared with SiHa-GFP cells (**Figure 3D**). These findings confirmed that TRIM31 promoted the malignant proliferation of cervical cancer cells.

### TRIM31 may act as a communicator between Snai2 and the Wnt/β-catenin pathway

**Figure 4:**
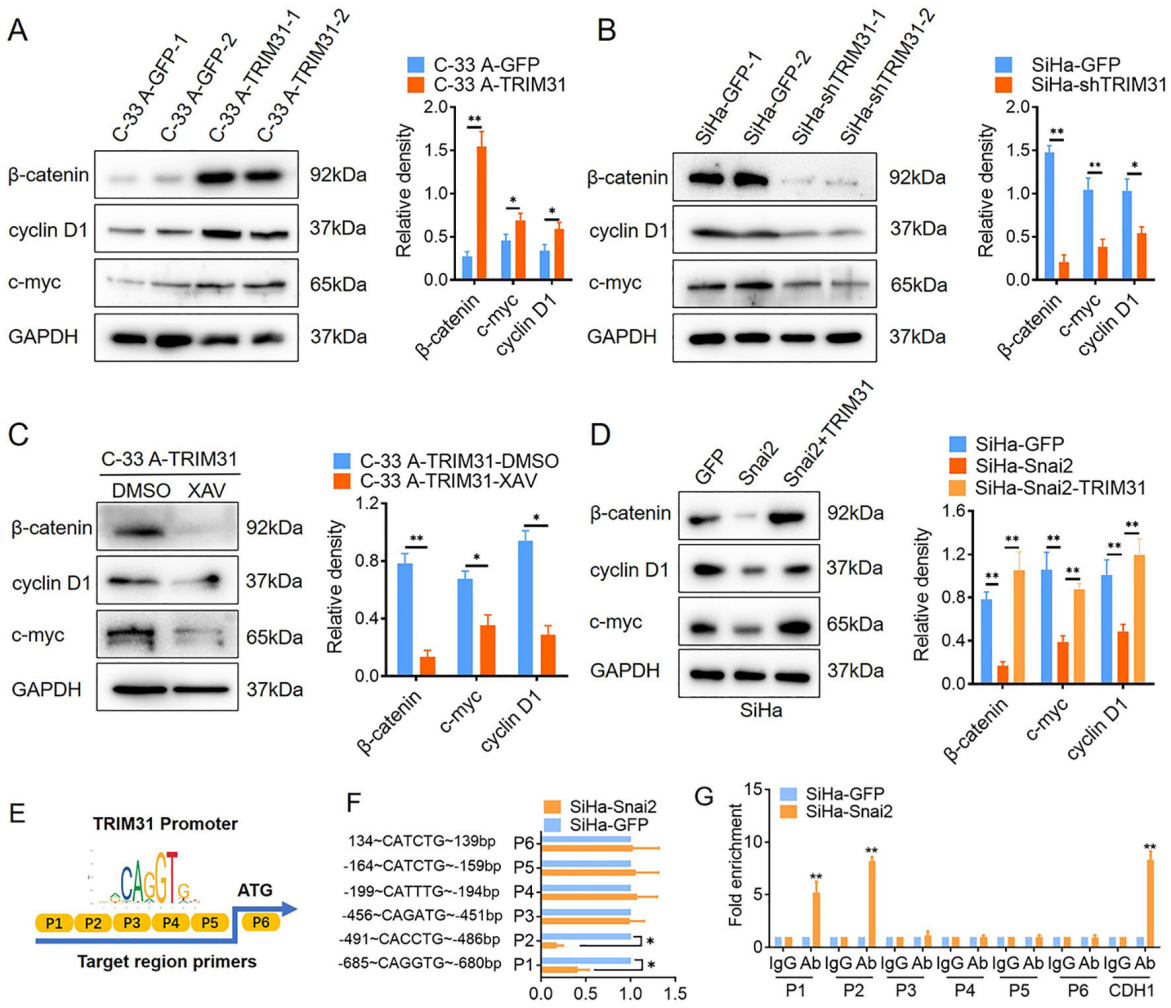
TRIM31 may communicate with Snai2 and the Wnt/β-catenin pathway. **(A)** Western blot of β-catenin, cyclin D1 and c-myc (downstream of Wnt/β-catenin) in C-33 A-TRIM31 and C-33 A-GFP cells with the analyzed relative density. **(B)** Western blot analysis of the relative density of β-catenin, cyclin D1 and c-myc (downstream of Wnt/β-catenin) in SiHa-shTRIM31 and SiHa-GFP cells. **(C)** Western blot of β-catenin, cyclin D1 and c-myc in C-33 A-TRIM31 cells treated with the Wnt/β-catenin pathway inhibitor XAV. **(D)** Western blot of β-catenin, cyclin D1 and c-myc in SiHa-Snai2 cells, which rescued TRIM31 expression. **(E)** Schematic representation of the reporter construct containing the firefly luciferase coding sequence fused to the TRIM31 promoter region, which was separated into 6 parts. **(F)** Luciferase reporter assays showing six TRIM31 promoter region fragments recognized by Snai2 in SiHa-Snai2 cells. **(G)** Chromatin immunoprecipitation showed that Snai2 bound to the E-box of the P1 and P2 fragments of the TRIM31 promoter region. *p < 0.05, **p < 0.01.

**Figure 5:**
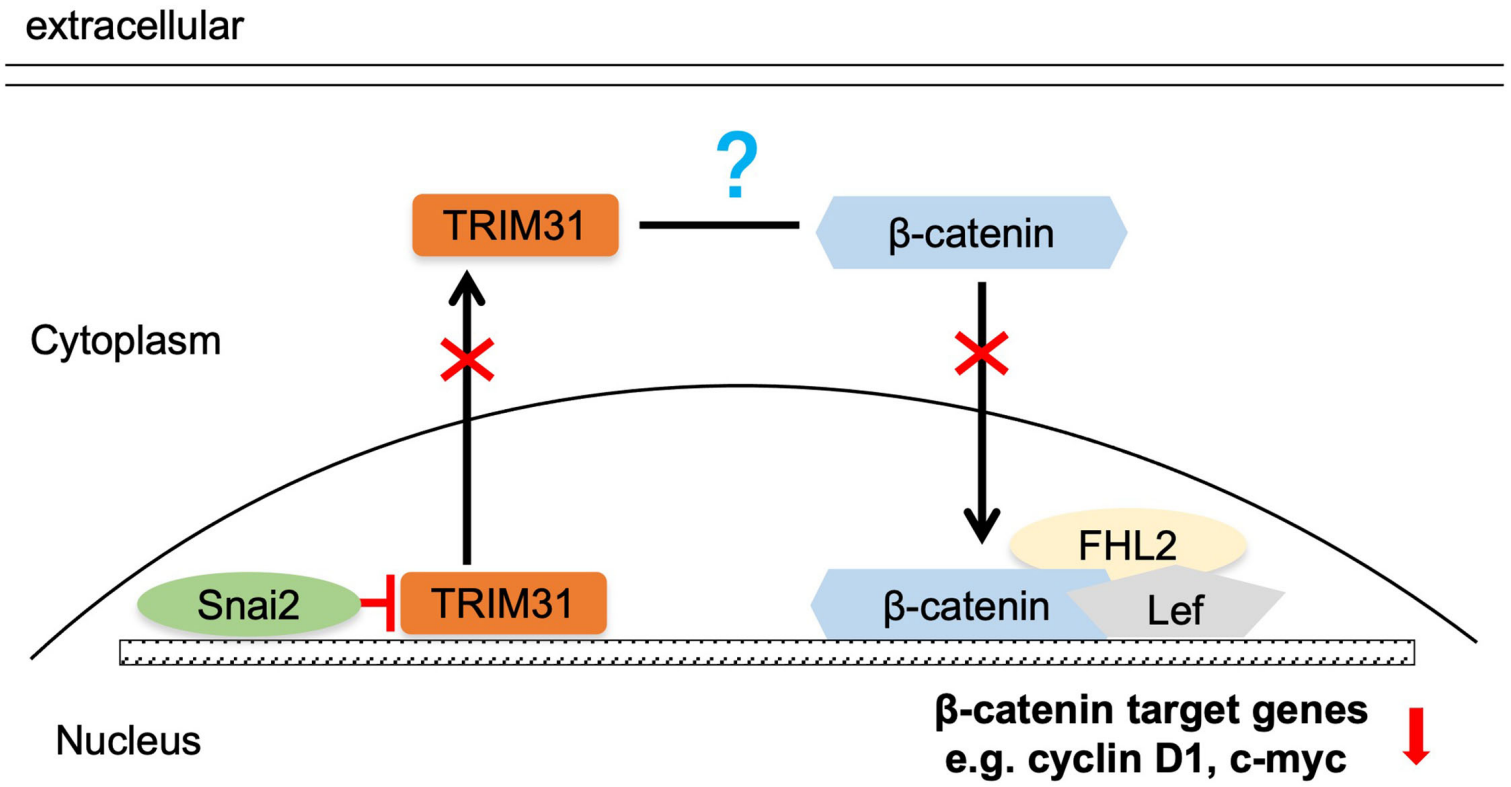
Summary of the mechanism by which Snai2 may impair Wnt/β-catenin pathway activity by transcriptionally inhibiting TRIM31.

Several studies have demonstrated that TRIM31 enhances the Wnt/β-catenin pathway during the oncogenesis and development of gastric cancer (GC) and acute myeloid leukemia (AML) (17, 18). Wnt/β-catenin pathway activity was examined in cervical cancer cells with aberrant expression of TRIM31, as mentioned above. The downstream proteins of the classical Wnt signaling pathway, β-catenin, cyclin D1 and c-myc, were significantly upregulated in C-33 A-TRIM31 cells compared with those in C-33 A-GFP cells (**Figure 4A**). In contrast, β-catenin, cyclin D1 and c-myc were significantly downregulated in SiHa-shTRIM31 cells compared with SiHa-GFP cells (**Figure 4B**). XAV, an inhibitor of the Wnt/β-catenin pathway, can simulate the degradation of β-catenin. The application of XAV to C-33 A-TRIM31 cells resulted in a significant reduction in β-catenin, cyclin D1 and c-myc levels (**Figure 4C**). These results suggested that the expression of TRIM31 could increase the activity of the Wnt/β-catenin pathway. Snai2 was proven to inhibit the activity of the Wnt/β-catenin pathway in our previous studies. Whether TRIM31 is a member of the Snai2-Wnt/β-catenin pathway axis needs to be confirmed. Furthermore, the levels of β-catenin, cyclin D1 and c-myc were restored when the expression of TRIM31 in SiHa-Snai2 cells was rescued (**Figure 4D**). These findings suggested that TRIM31 might communicate between Snai2 and the Wnt/β-catenin pathway. TRIM31 was regulated by Snai2 via a dual-luciferase reporter assay and ChIP for the next step. Snai2 can recognize and bind to the -680 to -491bp promoter region of TRIM31 (**Figures 4E-G**). These findings indicate a new possible mechanism by which Snai2 regulates the Wnt/β-catenin pathway by transcriptionally inhibiting TRIM31. However, the mechanism by which TRIM31 regulates the Wnt/β-catenin pathway remains to be further explored (**Figure 5**).

## Discussion

The ubiquitination process is catalyzed by E1, E2 and E3, among which E3 ubiquitin ligases are involved mainly in the recognition and binding of target proteins (19). TRIM31 functions as an E3 ubiquitin ligase that belongs to the TRIM family. This study is the first to confirm that TRIM31 is upregulated in cervical cancer and promotes the proliferation of cervical cancer cells (**Figures 2**, **3**). However, the clinical correlation of TRIM31 with cancer is still elusive. TRIM31 is upregulated in a variety of tumors, such as hepatocellular carcinoma, gallbladder cancer and colorectal cancer, and is associated with an aggressive phenotype, advanced disease status and poor prognosis (15, 16, 20). These findings suggest that the upregulation of TRIM31 is a common feature of many epithelial cancers and predicts a poor prognosis.

The effects of TRIM31 on the proliferation of cervical cancer cells were investigated, and TRIM31 significantly increased proliferation *in vitro* (**Figure 3**). Interestingly, TRIM31 promoted the expression of downstream genes of the Wnt/β-catenin pathway, whereas an inhibitor of the Wnt/β-catenin pathway blocked this ability (**Figures 4A-C**). TRIM31 regulates Wnt/β-catenin signaling to promote acute myeloid leukemia progression and sensitivity to daunorubicin (18). TRIM31 promotes gastric cancer cell proliferation and invasion through activating the Wnt/β-catenin pathway by regulating Axin1 protein stability (17). In gallbladder cancer, TRIM31 promotes proliferation and invasion via the PI3K/Akt signaling pathway. The PI3K/AKT/IKK alpha pathway regulates the activation of NF-kappa B and β-catenin in CRC cell lines (16, 21). In summary, similar with its role in other tumors, TRIM31 may promote the proliferation of cervical cancer cells through the classical Wnt pathway.

The negative correlation between Snai2 and TRIM31 was confirmed in our research (**Figure 1**), which has not been reported previously. This study reveal that Snai2 recognizes and binds to the E-box region of TRIM31 and regulates the expression of TRIM31 (**Figures 4E-G**). Snai2 was found to inhibit the Wnt/β-catenin signaling pathway in cervical cancer in our previous studies (22). Interestingly, TRIM31 promotes the expression of genes downstream of the Wnt/β-catenin pathway. Furthermore, when the expression of TRIM31 in SiHa-Snai2 cells was rescued, genes downstream of the Wnt/β-catenin pathway were recovered (**Figure 4D**). These findings suggest the possible role of TRIM31 in the mechanism by which Snai2 inhibits the Wnt/β-catenin pathway (**Figure 5**). TRIM31 has been proposed to be involved in several carcinogenic mechanisms, such as regulating the P53, mTORC1, PI3K-Akt, NF-kappa B and Wnt/β-catenin pathways to promote tumor onset and progression (23).

Although this study has demonstrated that TRIM31 is a tumor enhancer in cervical cancer cells and probably as a potential target factor of the transcription factor Snai2, further validation is still required to confirm the role of TRIM31 in promoting tumor growth in animal models and further investigate whether TRIM31 holds clinical significance in cervical cancer it would be more important. Despite our intention to analyze the correlation between TRIM31 and the survival of cervical cancer patients using online databases, regrettably, no significant positive results were obtained. We propose that the underlying reason may be associated with its dual role in cancer (tumor suppressor role or oncogene role) (23). Additionally, further studies are still needed to elucidate the molecular mechanisms by which TRIM31 regulates the activity of the Wnt/β-catenin signaling pathway. These studies will further elucidate the potential role of the Snai2-TRIM31-Wnt/β-catenin pathway axis in the progression of cervical cancer.

## Data Availability

The datasets presented in this study can be found in online repositories. The names of the repository/repositories and accession number(s) can be found in the article/**Supplementary Material**.

## References

[1] Bray F Ferlay J Soerjomataram I Siegel RL Torre LA Jemal A. Global cancer statistics 2018: GLOBOCAN estimates of incidence and mortality worldwide for 36 cancers in 185 countries. CA Cancer J Clin. (2018) 68:394–424. doi: 10.3322/caac.21492, PMID: 30207593

[2] Li K Li Q Song L Wang D Yin R. The distribution and prevalence of human papillomavirus in women in mainland China. Cancer. (2019) 125:1030–7. doi: 10.1002/cncr.32003, PMID: 30748006

[3] Yang L Shi P Zhao G Xu J Peng W Zhang J. Targeting cancer stem cell pathways for cancer therapy. Signal Transduct Target Ther. (2020) 5:8. doi: 10.1038/s41392-020-0110-5, PMID: 32296030 PMC7005297

[4] Cao HZ Liu XF Yang WT Chen Q Zheng PS. LGR5 promotes cancer stem cell traits and chemoresistance in cervical cancer. Cell Death Dis. (2017) 8:e3039. doi: 10.1038/cddis.2017.393, PMID: 28880275 PMC5636966

[5] Feng Q Li S Ma HM Yang WT Zheng PS. LGR6 activates the Wnt/β-catenin signaling pathway and forms a β-catenin/TCF7L2/LGR6 feedback loop in LGR6^high^ cervical cancer stem cells. Oncogene. (2021) 40:6103–14. doi: 10.1038/s41388-021-02002-1, PMID: 34489551 PMC8530990

[6] Liu SY Zheng PS. High aldehyde dehydrogenase activity identifies cancer stem cells in human cervical cancer. Oncotarget. (2013) 4:2462–75. doi: 10.18632/oncotarget.1578, PMID: 24318570 PMC3926841

[7] Hultgren NW Fang JS Ziegler ME Ramirez RN Phan DTT Hatch MMS. Slug regulates the Dll4-Notch-VEGFR2 axis to control endothelial cell activation and angiogenesis. Nat Commun. (2020) 11:5400. doi: 10.1038/s41467-020-18633-z, PMID: 33106502 PMC7588439

[8] Zhang Z Li L Wu C Yin G Zhu P Zhou Y. Inhibition of Slug effectively targets leukemia stem cells via the Slc13a3/ROS signaling pathway. Leukemia. (2020) 34:380–90. doi: 10.1038/s41375-019-0566-x, PMID: 31492896 PMC6995768

[9] Kahounová Z Remšík J Fedr R Bouchal J Mičková A Slabáková E. Slug-expressing mouse prostate epithelial cells have increased stem cell potential. Stem Cell Res. (2020) 46:101844. doi: 10.1016/j.scr.2020.101844, PMID: 32590255

[10] Lee Y Yoon J Ko D Yu M Lee S Kim S. TMPRSS4 promotes cancer stem-like properties in prostate cancer cells through upregulation of SOX2 by SLUG and TWIST1. J Exp Clin Cancer Res. (2021) 40:372. doi: 10.1186/s13046-021-02147-7, PMID: 34809669 PMC8607621

[11] Moon JH Lee SH Koo BS Kim JM Huang S Cho JH. Slug is a novel molecular target for head and neck squamous cell carcinoma stem-like cells. Oral Oncol. (2020) 111:104948. doi: 10.1016/j.oraloncology.2020.104948, PMID: 32771963

[12] Masuo K Chen R Yogo A Sugiyama A Fukuda A Masui T. SNAIL2 contributes to tumorigenicity and chemotherapy resistance in pancreatic cancer by regulating IGFBP2. Cancer Sci. (2021) 112:4987–99. doi: 10.1111/cas.15162, PMID: 34628696 PMC8645768

[13] Guo Y Li Q Zhao G Zhang J Yuan H Feng T. Loss of TRIM31 promotes breast cancer progression through regulating K48- and K63-linked ubiquitination of p53. Cell Death Dis. (2021) 12:945. doi: 10.1038/s41419-021-04208-3, PMID: 34650049 PMC8516922

[14] Guo P Ma X Zhao W Huai W Li T Qiu Y. TRIM31 is upregulated in hepatocellular carcinoma and promotes disease progression by inducing ubiquitination of TSC1-TSC2 complex. Oncogene. (2018) 37:478–88. doi: 10.1038/onc.2017.349, PMID: 28967907

[15] Wang H Yao L Gong Y Zhang B. TRIM31 regulates chronic inflammation via NF-κB signal pathway to promote invasion and metastasis in colorectal cancer. Am J Transl Res. (2018) 10:1247–59., PMID: 29736218 PMC5934584

[16] Li H Zhang Y Hai J Wang J Zhao B Du L. Knockdown of TRIM31 suppresses proliferation and invasion of gallbladder cancer cells by down-regulating MMP2/9 through the PI3K/Akt signaling pathway. BioMed Pharmacother. (2018) 103:1272–8. doi: 10.1016/j.biopha.2018.04.120, PMID: 29864908

[17] Feng Q Nie F Gan L Wei X Liu P Liu H. Tripartite motif 31 drives gastric cancer cell proliferation and invasion through activating the Wnt/β-catenin pathway by regulating Axin1 protein stability. Sci Rep. (2023) 13:20099. doi: 10.1038/s41598-023-47139-z, PMID: 37973999 PMC10654727

[18] Xiao Y Deng T Ming X Xu J. TRIM31 promotes acute myeloid leukemia progression and sensitivity to daunorubicin through the Wnt/β-catenin signaling. Biosci Rep. (2020) 40:BSR20194334. doi: 10.1042/BSR20194334, PMID: 32232394 PMC7160243

[19] Hershko A Heller H Elias S Ciechanover A. Components of ubiquitin-protein ligase system. Resolution, affinity purification, and role in protein breakdown. J Biol Chem. (1983) 258:8206–14. doi: 10.1016/S0021-9258(20)82050-X, PMID: 6305978

[20] Guo P Qiu Y Ma X Li T Ma X Zhu L. Tripartite motif 31 promotes resistance to anoikis of hepatocarcinoma cells through regulation of p53-AMPK axis. Exp Cell Res. (2018) 368:59–66. doi: 10.1016/j.yexcr.2018.04.013, PMID: 29665353

[21] Agarwal A Das K Lerner N Sathe S Cicek M Casey G. The AKT/I kappa B kinase pathway promotes angiogenic/metastatic gene expression in colorectal cancer by activating nuclear factor-kappa B and beta-catenin. Oncogene. (2005) 24:1021–31. doi: 10.1038/sj.onc.1208296, PMID: 15592509

[22] Cui N Yang WT Zheng PS. Slug inhibits the proliferation and tumor formation of human cervical cancer cells by up-regulating the p21/p27 proteins and down-regulating the activity of the Wnt/β-catenin signaling pathway via the trans-suppression Akt1/p-Akt1 expression. Oncotarget. (2016) 7:26152–67. doi: 10.18632/oncotarget.8434, PMID: 27036045 PMC5041971

[23] Guo Y Lin P Hua Y Wang C. TRIM31: A molecule with a dual role in cancer. Front Oncol. (2022) 12:1047177. doi: 10.3389/fonc.2022.1047177, PMID: 36620540 PMC9815508

