# Supplementary figures and images for: TRIM31 acts as an intermediate molecule in the process by which Snai2 impairs the proliferation of cervical cancer cells
Source: Front Oncol. 2025 Aug 22;15:1537991. doi: 10.3389/fonc.2025.1537991 (PMC12411157; doi:10.3389/fonc.2025.1537991)

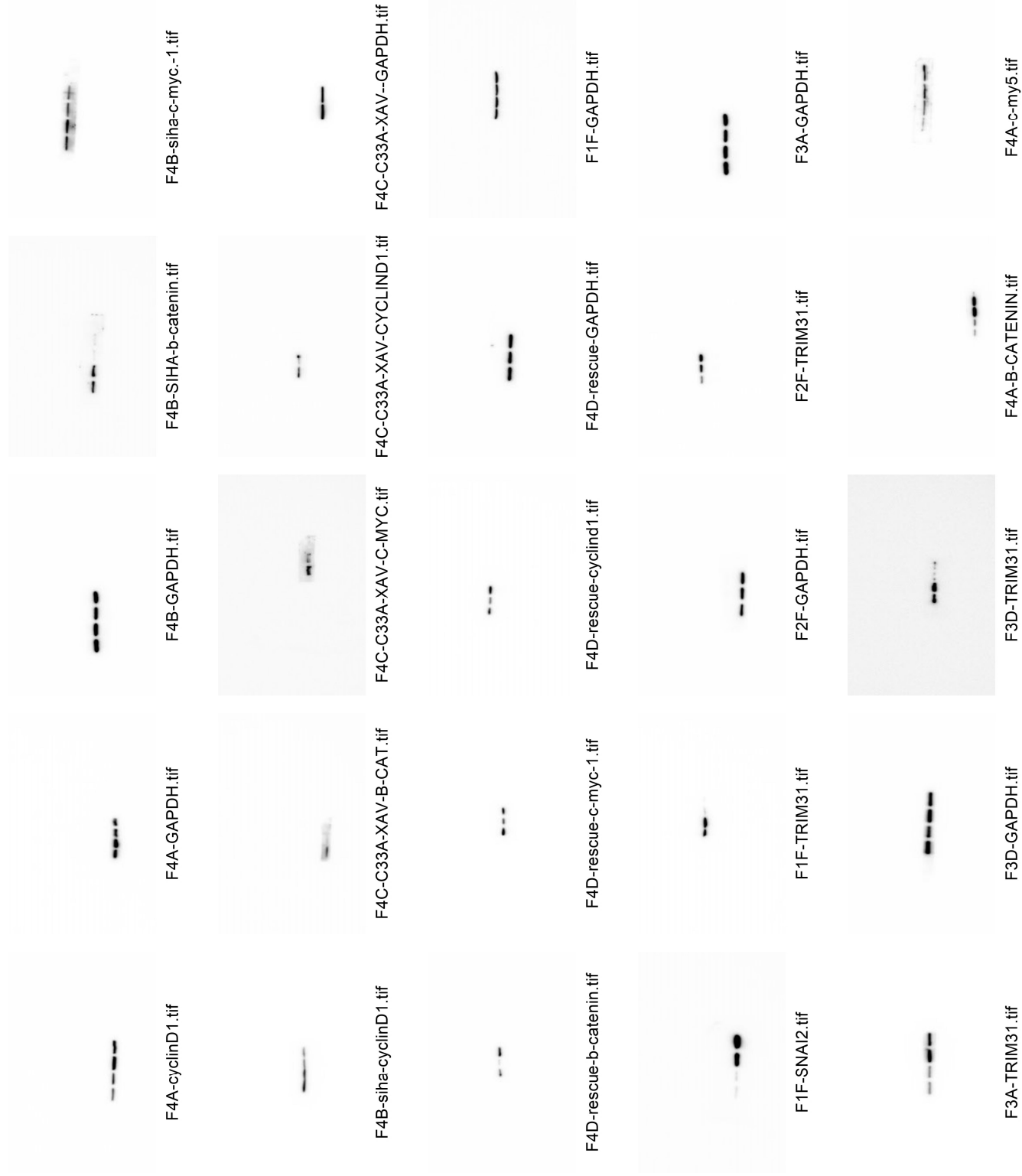

Supplement: Supplementary file 1 [file DataSheet1.pdf]
