# Supplementary material for: TRIM31 acts as an intermediate molecule in the process by which Snai2 impairs the proliferation of cervical cancer cells
Source: Front Oncol. 2025 Aug 22;15:1537991. doi: 10.3389/fonc.2025.1537991 (PMC12411157; doi:10.3389/fonc.2025.1537991)
Supplement: Supplementary file 2 [file Table1.doc]

**Supplement Information**

**Table S1**. The list of primer sequences that used for luciferase assays in this study.

| **Primer name** | **F/R** | **Sequence** |
| --- | --- | --- |
| **P1** | F | GAAGATCTCAAAGAATGGGCACGGA |
|  | R | CGACGCGTTTGTTTTTGCACTTTGC |
| **P2** | F | GAAGATCTCACAAAATTCTGTGAGG |
|  | R | CGACGCGTAAGGGCAATTCTCTA |
| **P3** | F | GAAGATCTTAGAGAATTGCCCTT |
|  | R | CGACGCGTGGTCACAGCCAGTTCA |
| **P4** | F | GAAGATCTGTTCAGGACAACTTATT |
|  | R | CGACGCGTTTGCTACCTCTTGAG |
| **P5** | F | GAAGATCTTCAAGAGGTAGCAT |
|  | R | CGACGCGTAGCAGAGAGAGAG |
| **P6** | F | GAAGATCTTGTTGTTCTGTCAT |
|  | R | CGACGCGTATTTGAGGCAGAAAT |

**Table S2. The list of primer sequences that used for chromatin immunoprecipitation assay (ChIP)** in this study.

| **Primer name** | **F/R** | **Sequence** |
| --- | --- | --- |
| **P1** | F | CAAAGAATGGGCACGGAG |
|  | R | GCCGTTTTTGCACTTTGCTC |
| **P2** | F | CACAAAATTCTGTGAGG |
|  | R | AAGGGCAATTCTCTA |
| **P3** | F | GGTAGAGAATTGCCCTTCC |
|  | R | CCGGTCACAGCCAGTTCA |
| **P4** | F | GTTCAGGACAACTTATTCCGC |
|  | R | CCTTGCTACCTCTTGAGAAC |
| **P5** | F | TCAAGAGGTAGCATGGT |
|  | R | GAAGAGCAGAGAGAGAG |
| **P6** | F | TGTTGTTCTGTCATGGCC |
|  | R | GTGATGCATTTGAGGCAG |
| **E-cadherin** | F | CGTCGGAACTGCAAAGC |
|  | R | TATGTGCGGTCGGGTCG |
| **3’UTR** | F | TTGAAGATTATAGAAGAAGG |
|  | R | TAACAAACTCATGACCTTC |
